# Heart rate variability in children with type 1 diabetes mellitus

**DOI:** 10.1590/0103-0582201432215513

**Published:** 2014-06

**Authors:** Camila Balsamo Gardim, Bruno Affonso P. de Oliveira, Aline Fernanda B. Bernardo, Rayana Loch Gomes, Francis Lopes Pacagnelli, Roselene Modolo R. Lorençoni, Luiz Carlos M. Vanderlei

**Affiliations:** 1 Faculdade de Ciências e Tecnologia da Unesp, Presidente Prudente, SP, Brasil; 2 Universidade de São Paulo (USP), Ribeirão Preto, SP, Brasil; 3 Universidade do Oeste Paulista (Unoeste), Presidente Prudente, SP, Brasil

**Keywords:** diabetes mellitus, type 1, child, autonomic nervous system, heart rate variability

## Abstract

**OBJECTIVE:**

To gather current information about the effects of type 1 diabetes mellitus on children's cardiac autonomic behavior.

**DATA SOURCES:**

The search of articles was conducted on PubMed, Ibecs, Medline, Cochrane, Lilacs, SciELO and PEDro databases using the MeSH terms: "autonomic nervous system", "diabetes mellitus", "child", "type 1 diabetes mellitus", "sympathetic nervous system" and "parasympathetic nervous system", and their respective versions in Portuguese (DeCS). Articles published from January 2003 to February 2013 that enrolled children with 9-12 years old with type 1 diabetes mellitus were included in the review.

**DATA SYNTHESIS:**

The electronic search resulted in four articles that approached the heart rate variability in children with type 1 diabetes mellitus, showing that, in general, these children present decreased global heart rate variability and vagal activity. The practice of physical activity promoted benefits for these individuals.

**CONCLUSIONS:**

Children with type 1 diabetes mellitus present changes on autonomic modulation, indicating the need for early attention to avoid future complications in this group.

## Abstract (es)

**OBJETIVO:**

Reunir informaciones actuales sobre los efectos de la diabetes mellitus tipo 1 sobre el comportamiento cardiaco de niños.

**FUENTES DE DATOS:**

La búsqueda de artículos se realizó en las bases de datos PubMed, Ibecs, Medline, Cochrane, Lilacs y PEDro por medio de los descriptores del área de salud (DeCS) a continuación: "sistema nervioso autónomo", "diabetes mellitus", "niño", "diabetes mellitus tipo 1", "sistema nervioso simpático" y "sistema nervioso parasimpático" sus respectivas versiones en lengua inglesa (MeSH).Los artículos fueron publicados de enero de 2003 a febrero de 2013, implicando a niños de nueve a 12 años portadores de diabetes mellitus tipo 1.

**SÍNTESIS DE LOS DATOS:**

La búsqueda resultó en cuatro artículos que trataban la variabilidad de la frecuencia cardiaca en niños con diabetes mellitus tipo 1, demostrando que, por lo general, esos niños presentan reducción de la variabilidad de la frecuencia cardiaca global y de la actividad vagal. La práctica de actividad física promueve beneficios al organismo de niños con diabetes mellitus tipo 1.

**CONCLUSIONES:**

Niños diabéticos tipo 1 presentan modificaciones en la modulación autonómica, lo que demuestra la necesidad de atención temprana a esa población para evitar complicaciones futuras.

## Introduction

Type 1 diabetes mellitus (T1DM) is considered one of the most important chronic diseases in childhood worldwide and is a major challenge to health systems all over the world^(^ 1 ^,^ 2 ^)^. 

Data from 1985 estimated 30 million people with diabetes mellitus (DM) in the world and this number rose to 173 million in 2002, projected to reach 300 million in 2030^(^ 3 ^)^. Of this total, only 5-10% represent the population with T1DM^(^ 4 ^)^. Furthermore, in Brazil, it is estimated that the prevalence and incidence of T1DM in individuals under 14 years are from 4/10 thousand and 8/100 thousand inhabitants, respectively, and the trend is that there is an increase in these numbers due to new lifestyle habits in childhood as a result of technological development and urbanization^(^ 5 ^)^.

T1DM promotes several consequences of micro- and macrovascular changes that lead to dysfunctions and failure in different organs. Among the acute complications stand out: diabetic ketoacidosis, hypoglycemia, and seizures^ (6-8)^. Among chronic complications, we emphasize nephropathy, retinopathy, arthropathy, neuropathy, and autonomic neuropathy^(^ 7 ^-^ 9 ^)^.

Among these complications, diabetic autonomic neuropathy is poorly understood and recognized, despite the significant damage to the autonomic nervous system^(^ 6 ^,^ 9 ^)^, which plays and important role in regulating physiological processes of the human body in both normal and pathological conditions^(^ 6 ^,^ 10 ^,^ 11 ^)^.

Of the existing ways to assess the behavior of the autonomic nervous system is through heart rate variability (HRV), a simple and noninvasive method that describes variations in the intervals between consecutive heart beats (R-R intervals)^(12) ^and evaluates the behavior of the nervous system effectively on various situations, including pathological^(^ 12 ^-^ 16 ^)^. HRV can be analyzed with the use of indexes obtained by linear methods in the time and frequency domains (Table 1)^(^ 11 ^,^ 17 ^)^.

**Table 1:**
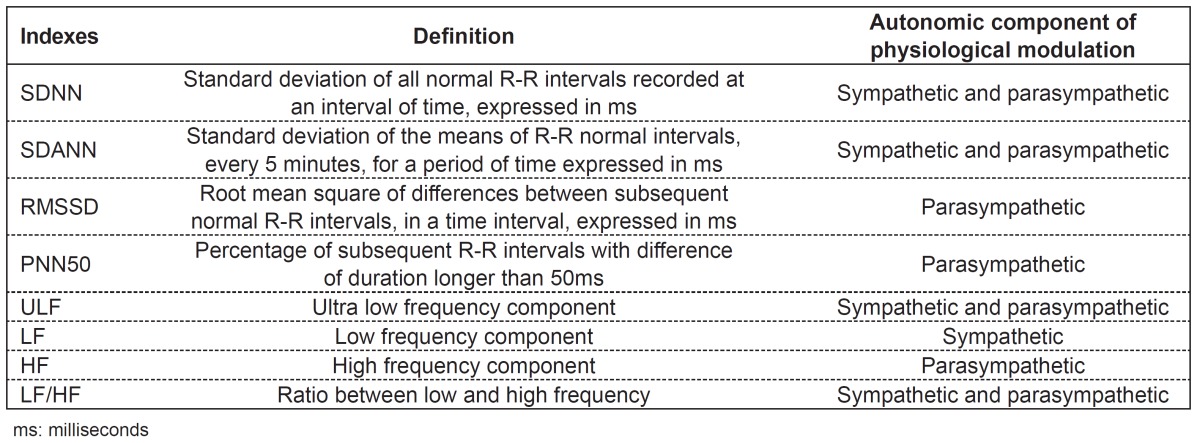
Linear indexes of heart rate variability, meaning, and autonomic components of the physiological modulation

Changes in patterns of HRV provide a sensitive and early indicator of impaired health. High HRV is a sign of good adaptation, characterizing a healthy individual with efficient autonomic mechanisms. Inversely, low HRV is generally an indicator of abnormal and insufficient adaptation of the autonomic nervous system, which may indicate the presence of physiological malfunction in the individual^(^ 17 ^)^. Reduced HRV has been identified as a strong indicator related to adverse effects in normal individuals and in patients with many diseases, reflecting the vital role that the autonomic nervous system plays in maintaining health^(^ 14 ^,^ 17 ^)^. Decreased vagal activity, which promotes a reduction in HRV, is associated with increased risk for morbidity and mortality from all causes and with the development of various risk factors^(^ 18 ^)^. Therefore, the use of this tool may represent an important predictor of the behavior of the autonomic nervous system prior to the installation of the complications of a chronic disease, being a means to better understand its causes and to predict cardiovascular and metabolic health, as well as to plan preventive treatments, assess the evolution of the pathological condition, and to monitor therapeutic procedures in the long run in order to verify its effectiveness. 

Several studies that assessed T1DM through HRV were found in the literature, mostly in adults and in the elderly^(^ 19 ^-^ 22 ^)^. However, the search in the pertinent literature showed a lack of studies that analyze the autonomic modulation in children with T1DM using HRV as a means of analysis. T1DM leads to irreversible complications to these children's bodies, impairing growth in the behavioral, somatic, and social spheres, besides decreasing the sensitivity of autonomic reflexes, being partly responsible for the reduction in life expectancy^(^ 23 ^)^.

Therefore, this study aimed to gather current information regarding the effects of T1DM on cardiac autonomic behavior of children in order to contribute to a better understanding for researchers and clinicians who work with this theme.

## Method

The search for the articles used in this study occurred from March to April 2013 through searches in the databases Medical Literature Analysis and Retrieval System Online (Medline/PubMed), Physiotherapy Evidence Database (PEDro), Scientific Electronic Library Online (SciELO), *Literatura Latino-Americana e do Caribe em Ciências da Saúde* (Lilacs), The Cochrane Library (Cochrane), and Índice Bibliográfico Espanhol em Ciências da Saúde (Ibecs), considering the articles published from January 2003 to February 2013.

For the search, the following keywords were used: "autonomic nervous system", "diabetes mellitus", "children", "type 1 diabetes mellitus", "sympathetic nervous system" and "parasympathetic nervous system". The terms in Portuguese were defined based on the Health Science Descriptors Headings (*Descritores em Ciências da Saúde - DeCS)* and its English correspondents, on the Medical Subject Headings (MeSH). We also used in the searches not the descriptor, but the keyword of this study, "heart rate variability", and its equivalent in the English language.

With these words, the following intersections were performed: autonomic nervous system and diabetes mellitus and children; diabetes mellitus and autonomic nervous system; type 1 diabetes mellitus and autonomic nervous system; diabetes mellitus and heart rate variability; diabetes mellitus and sympathetic nervous system; and diabetes mellitus and parasympathetic nervous system.

As inclusion criteria, we considered the articles published from January 2003 to February 2013 in Portuguese, English and Spanish, covering all types of study design that used children aged from 9 to 12 years with T1DM and that addressed the influence of T1DM on the autonomic nervous system, assessed by HRV. We excluded from the search studies such as dissertations, theses, and editorials.

For the selection, the studies initially underwent a screening of titles held by a single researcher. We selected the titles that presented as the main idea the following aspects: the influence of diabetes on the autonomic nervous system, heart rate variability in children with DM, the effects of diabetes on the health of these children, and titles that brought evidence on the issue and the changes that DM promoted in sympathetic and parasympathetic components of the autonomic nervous system in children. After this initial selection, we carried out a filtration, which excluded duplicate titles, since the articles were selected from different databases.

Then, all titles chosen had their abstracts read and studied in detail in order to select the articles that focused on children, the influence of diabetes on the autonomic nervous system, assessed by HRV. After excluding abstracts that were inadequate to the topic, we performed a full-text analysis and considered those who met the inclusion criteria. Furthermore, all selected abstracts had their references checked to identify studies that were not found in the first electronic search. All steps were followed by a senior reviewer, who conducted the final judgment of inclusion of articles.

Data were described qualitatively and tabulated according to the following characteristics: authors, year of publication, objectives, HRV indexes analyzed, and conclusions obtained on each article.

## Results

In the search and electronic selection strategy, 1,687 titles were found. Among the selected articles, 1,495 references that did not address the topic were eliminated. We read the abstracts of the 192 remaining articles and, of this total, we eliminated another 154 titles because they were repeated. At the end, we selected 38 texts for full analysis, which led to the exclusion of other 34 references for not presenting the age group, totaling four final articles.

Table 2 shows the final four articles resulting from the electronic search. None of the studies was randomized or semi-randomized. One of the studies was observational, descriptive, and cross-sectional^(^ 24 ^)^, two were case-control, observational, and cross-sectional^(^ 16 ^,^ 25 ^)^ and the other was case-control, observational and longitudinal^(^ 26 ^)^.

**Table 2:**
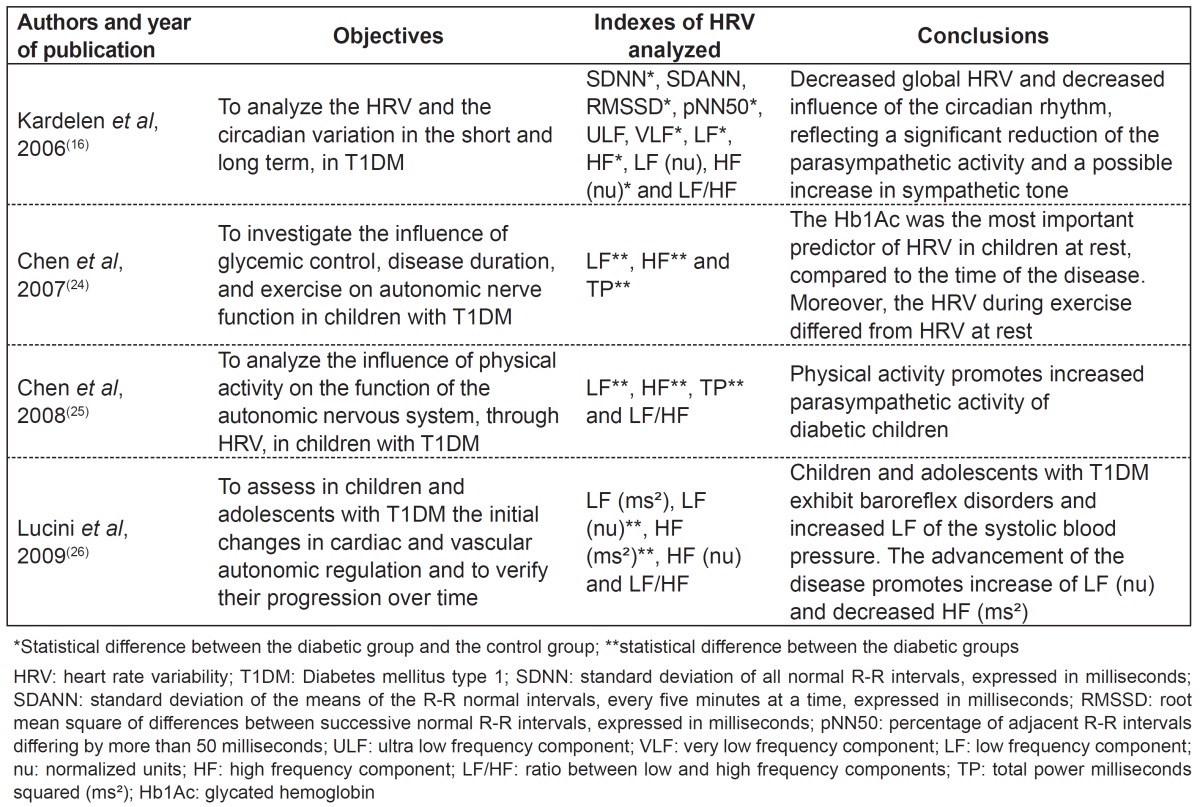
Description of the studies on the influence of type 1 diabetes mellitus on the autonomic nervous system, according to authors/year, population, analyzed indexes, and conclusions

### Observational, descriptive and cross-sectional study

Chen et al^(^ 24 ^)^, using the low frequency component - LF (in milliseconds squared - ms²), high frequency component - HF (ms²) and total power - TP (ms²) indexes, investigated the influence of glycemic control (Hb1Ac), duration of the T1DM, and of physical exercise on the autonomic modulation of children with T1DM. The analyzed children were divided into four groups: Group 1 (n=16; Hb1Ac ≤8%; age: 9.6±1.0 years; duration of T1DM: 2.1±1.2 years), Group 2 (n=15; Hb1Ac ≤8%; age: 9.7±1.5 years; duration of T1DM: 6.5±1.4 years), Group 3 (n=21; Hb1Ac >8%; age: 10.1±1.2 years; duration of T1DM: 2.4±1.2 years) and Group 4 (n=27; Hb1Ac >8%; age: 10.6±1.4 years; duration of T1DM: 6.8±1.8 years). To participate in the study, the children could not present *Acantose Nigricans*, hematological diseases, Turner's or Prader-Willi syndromes, and they could not take steroids or growth hormones.

Authors observed that, at rest, Hb1Ac and the duration of the disease were negatively correlated with all indexes of HRV. Both Hb1Ac and the duration of the disease were significant predictors for the TP index (ms²); however, only the Hb1Ac was a significant predictor for the LF (ms²) and HF (ms²) indexes. Children with T1DM who presented Hb1Ac higher than 8% and duration of the disease above 4.5 years had a lower HRV. For the exercise protocol, the children remained 10 minutes at rest and, then performed the exercise for 10 minutes with a steady rhythm on the stepper (JK-355c; JKexer, Taipei, Taiwan). The electrocardiogram (ECG) was continuously monitored throughout all test to obtain the indexes of HRV. Lower values ​​for all the studied indexes were observed during exercise in all groups, indicating that glycemic control and disease duration do not influence HRV during the exercise. 

### Case-control, observational and cross-sectional studies

We found two studies of this type. Kardelen et al^(16) ^investigated, in children with T1DM, the autonomic modulation by means of the HRV indexes analyzed in the time (SDNN, SDANN, RMSSD and pNN50) and frequency (ULF, VLF, LF, HF and LF/HF ratio) domains. We analyzed data from 93 children distributed between the Diabetic Group (n=47; age: 12.0±4.0 years; duration of T1DM: 4.2±3.2 years) and the Control Group (n=46; age: 10.8±3.1 years). To participate in the study, children could not: have a history of cardiac and/or respiratory disease, need medication, or have illness or consumption of drugs known to affect heart rate. 

The authors observed a reduction of HRV indexes both in the time domain, except for the SDANN, and the frequency domain, with marked reduction in the values of VLF, LF and HF. The authors also evaluated the circadian rhythm of HRV and observed differences between groups. All indexes of the parasympathetic and sympathetic tone assessed over a 24-hour period increased significantly overnight. In diabetic children with autonomic neuropathy, the demonstration of the reduction in the increase of parasympathetic tone protection at night led to the hypothesis that nocturnal predominance of sympathetic activity predisposes diabetic patients to cardiovascular events at any time. These changes in HRV indexes reflect a significant reduction in parasympathetic activity and, possibly, increased sympathetic activity. Furthermore, the authors observed that the magnitude of the influence of circadian rhythm is lower in diabetic children compared to normal children.

An article published by Chen et al^(^ 25 ^)^ explored the influence of the level of physical activity by means of a specific questionnaire on the role of the autonomic nervous system in children with T1DM. The children analyzed were divided into: Diabetic Group (n=93; age: 10.3±1.6 years; duration of T1DM: 4.1±2.1 years) and Control Group (n=107; age: 10.4±1.6 years). To participate in the study, the children could not have current of past clinical evidence of cardiovascular or neurological disease or make use of medications that could alter cardiovascular function or the activity of the autonomic nervous system. The results showed that, at rest, the LH (ms²), HF (ms²) and TP (ms²) indexes were lower in diabetics who presented low physical activity. Children with T1DM with moderate to intense physical activity did not differ from the healthy group. The authors concluded that children with T1DM should be encouraged to practice physical activities, which can benefit the function of the autonomic nervous system.

### Longitudinal, observational, case-control study

Lucini et al^(26) ^analyzed the autonomic modulation in children and adolescents with T1DM in order to assess the initial changes in cardiac and vascular autonomic regulation and check their progress over time. Children and adolescents were divided into four groups: Group of Healthy Children (n=32; age: 11.2±0.5 years), Group of Diabetic Children (n=46; age: 11.5±0.4 years; T1DM duration: 4.8±0.2 years), Group of Healthy Teenagers (n=36; age: 20.2±0.3 years), and Group of Diabetic Adolescents (n=47; age: 19.3±0.2 years; duration of T1DM: 10.3±0.7 years). As inclusion criteria, children and adolescents could not: present any concomitant disease (except for diabetes), make use of any medication (except for insulin), smoke, drink, or eat abusive amounts of food. The authors showed that children and adolescents with T1DM presented autonomic changes characterized by reduction of baroreceptor activity and increased LF of systolic blood pressure, indicating a lower HRV. The authors also assessed this population during a year and observed negative progression in the autonomic changes, with reduction of baroreceptor activity and reduction in the values of the LF (in normalized units - nu) and HF (ms²) indexes.

## Discussion

In general, analyzes of selected texts showed that: 1) children with T1DM presented reduction of the global HRV global and of the vagal activity compared to control children; 2) the practice of physical activity promotes benefits in autonomic modulation in children with T1DM.

Corroborating the findings of Kardelen et al^(^ 16 ^)^, Jaiswal et al, in a study published in 2013, also observed a reduction of overall HRV in young people with T1DM and mean age of 18±8.0 years^(^ 27 ^)^. The authors suggest that this reduction may be caused by diabetic autonomic neuropathy, which causes changes in physiological processes in the body, especially in the autonomic nervous system.

Higher global HRV global is related to a better condition of the autonomic nervous system and increased efficiency of responses to both internal and external stimuli to the body. In contrast, the reduction in global HRV, as it occurs in children with T1DM, is an indicative of abnormal and insufficient adaptation of the autonomic nervous system and physiological malfunction^(^ 27 ^,^ 28 ^)^. It has been found that the HRV is an effective method to detect autonomic changes in children with T1DM, allowing better discrimination between normal and abnormal physiology of these children. The presence of T1DM may lead to the loss of quality of life, increased risk of morbidity and mortality due to relevant changes in the autonomic nervous system, and to prolonged exposure to high glucose levels^(^ 29 ^)^. 

Considering the various changes introduced by T1DM in children, we highlight the importance of an adequate treatment, seeking healthy conditions for development, growth, and prevention of comorbidities. In this context, the practice of physical activity - which provides improvement in glucose uptake by the tissues independently of insulin, increased permeability of the cytoplasmic membrane, and augmentation of the action of the hormone, reducing the amount of medication and complications caused by DM^(^ 30 ^)^ - was highlighted in the literature. The article published by Chen et al^(^ 25 ^)^ explored the influence of physical activity on the function of the autonomic nervous system in children with T1DM. To assess the level of physical activity, the authors used a specific questionnaire; however, studies evaluating the effect of physical activity on cardiovascular conditioning in a direct manner are needed to confirm the importance of the practice in the autonomic control in this population. 

Other results, also published by Chen et al^(^ 24 ^)^ involving physical exercise, showed no influence of glycemic control or of the duration of the disease on the HRV during the exercise. However, the individuals with T1DM who presented Hb1Ac higher than 8% and disease duration above 4.5 had a lower HRV. The use of only one test of Hb1Ac as indicator of glycemic control may be considered a limitation of the study.

Finally, some considerations are noteworthy. None of the studies found were randomized clinical trials, and were not combined in the form of meta-analysis, which demonstrates the low level of scientific evidence of the theme and opens perspectives for studies in the area. Regarding methodological aspects of the studies analyzed, the environmental conditions in which the autonomic assessments were performed (such as temperature and humidity) were not described and there is no mention as to whether the analyzed children performed some kind of physical exercise regularly. 

In summary, the studies showed that children with T1DM exhibit changes in autonomic modulation characterized by the global reduction in HRV and in the vagal activity. Furthermore, it should be highlighted that the practice of any physical activity was beneficial to these individuals, as it promoted increased vagal activity.
