# Supplementary material for: Heart rate variability in children with type 1 diabetes mellitus
Source: Rev Paul Pediatr. 2014 Jun;32(2):279–85. doi: 10.1590/0103-0582201432215513 (PMC4183025; doi:10.1590/0103-0582201432215513)
Supplement: Translation

# Translation: Variabilidade da frequência cardíaca em crianças com diabetes melito tipo 1

**Authors:** Camila Balsamo Gardim, Bruno Affonso P. de Oliveira, Aline Fernanda B. Bernardo, Rayana Loch Gomes, Francis Lopes Pacagnelli, Roselene Modolo R. Lorençoni, Luiz Carlos M. Vanderlei

**Affiliations:** 1 Faculdade de Ciências e Tecnologia da Unesp, Presidente Prudente, SP, Brasil; 2 Universidade de São Paulo (USP), Ribeirão Preto, SP, Brasil; 3 Universidade do Oeste Paulista (Unoeste), Presidente Prudente, SP, Brasil

**Keywords:** diabetes mellitus tipo 1, criança, sistema nervoso autônomo, variabilidade da frequência cardíaca

## Abstract

**OBJETIVO:**

Reunir informações atuais acerca dos efeitos do diabetes melito tipo 1 sobre o comportamento autonômico cardíaco de crianças.

**FONTES DE DADOS:**

A busca dos artigos foi realizada nas bases de dados PubMed, Ibecs, Medline, Cochrane, Lilacs, SciELO e PEDro por meio dos seguintes descritores da área da saúde (DeCS): "sistema nervoso autônomo", "diabetes mellitus", "criança", "diabetes mellitus tipo 1", "sistema nervoso simpático" e "sistema nervoso parassimpático" e suas respectivas versões em língua inglesa (MeSH). Os artigos foram publicados de janeiro de 2003 a fevereiro de 2013, envolvendo crianças de nove a 12 anos portadoras de diabetes melito tipo 1.

**SÍNTESE DOS DADOS:**

A busca resultou em quatro artigos que abordam a variabilidade da frequência cardíaca em crianças com diabetes melito tipo 1, demonstrando que, em geral, essas crianças apresentam redução da variabilidade da frequência cardíaca global e da atividade vagal. A prática de atividade física promove benefícios no organismo de crianças com diabetes melito tipo 1.

**CONCLUSÕES:**

Crianças diabéticas tipo 1 apresentam modificações na modulação autonômica, o que demonstra a necessidade de atenção precoce a essa população para evitar complicações futuras.

## Introdução

O diabetes melito tipo 1 (DM1) é considerado uma das mais importantes doenças crônico-degenerativas da infância em esfera mundial e um grande desafio para os sistemas de saúde de todo o mundo^(^ ^1^ ^,^ ^2^ ^)^. 

 Dados de 1985 estimavam 30 milhões de pessoas com diabetes mellito (DM) no mundo e esse número cresceu para 173 milhões em 2002, com projeção de chegar a 300 milhões em 2030^(^ ^3^ ^)^. Desse total, apenas 5-10% representam a população com DM1^(^ ^4^ ^)^. Além disso, no Brasil, estima-se que a prevalência e a incidência do DM1 em indivíduos menores de 14 anos sejam de 4/10 mil e 8/100 mil habitantes, respectivamente, e a tendência é de crescimento nesses números devido aos novos hábitos de vida na infância, como consequência do avanço tecnológico e da urbanização^(^ ^5^ ^)^.

O DM1 promove diversas consequências resultantes de alterações micro e macrovasculares que levam a disfunções e insuficiências em diferentes órgãos. Dentre as complicações agudas, destaca-se a cetoacidose diabética, a hipoglicemia e a convulsão^(^ ^6^ ^-^ ^8^ ^)^. Dentre as complicações crônicas, ressaltam-se a nefropatia, a retinopatia, a artropatia, a neuropatia periférica e a neuropatia autonômica diabética^(^ ^7^ ^-^ ^9^ ^)^.

Dessas complicações, a neuropatia autonômica diabética é pouco entendida e reconhecida, apesar dos significantes prejuízos ao sistema nervoso autônomo^(^ ^6^ ^,^ ^9^ ^)^, o qual desempenha um papel importante na regulação dos processos fisiológicos do organismo humano, em condições tanto normais quanto patológicas^(^ ^6^ ^,^ ^10^ ^,^ ^11^ ^)^.

Uma das formas existentes para avaliar o comportamento do sistema nervoso autônomo é a variabilidade da frequência cardíaca (VFC), um método simples e não invasivo que descreve oscilações nos intervalos entre batimentos cardíacos consecutivos (intervalos R-R)^(12) ^e avalia com eficácia o comportamento do sistema nervoso autônomo diante de diversas situações, inclusive patológicas^(^ ^12^ ^-^ ^16^ ^)^. Para analisar a VFC, podem-se utilizar índices obtidos por meio de métodos lineares, nos domínios do tempo e da frequência (Quadro 1)^(^ ^11^ ^,^ ^17^ ^)^.

**Quadro 1:** Índices lineares de variabilidade da frequência cardíaca, significado e componentes autonômicos da modulação fisiológica ms: milissegundos

Mudanças nos padrões da VFC fornecem um indicador sensível e antecipado de comprometimento da saúde. Alta VFC é sinal de boa adaptação, caracterizando um indivíduo saudável com mecanismos autonômicos eficientes. Inversamente, a baixa VFC é, em geral, um indicador de adaptação anormal e insuficiente do sistema nervoso autônomo, o que pode indicar a presença de mau funcionamento fisiológico no indivíduo^(^ ^17^ ^)^. A redução da VFC tem sido apontada como um forte indicador de risco relacionado a eventos adversos em indivíduos normais e em pacientes com grande número de doenças, refletindo o papel vital que o sistema nervoso autônomo desempenha na manutenção da saúde^(^ ^14^ ^,^ ^17^ ^)^. A redução na atividade vagal, que promove redução da VFC, associa-se ao aumento do risco para morbidade e mortalidade por todas as causas e ao desenvolvimento de vários fatores de risco^(^ ^18^ ^)^. Portanto, a utilização dessa ferramenta pode representar um importante preditor do comportamento do sistema nervoso autônomo antes da instalação das complicações de uma doença crônica, sendo um meio para melhor entender suas causas e predizer a saúde cardiovascular e metabólica, bem como para planejar tratamentos preventivos, avaliar a evolução do quadro patológico e monitorar procedimentos terapêuticos em longo prazo, a fim de verificar sua eficácia. 

Diversos estudos que avaliaram o DM1 por meio da VFC foram encontrados na literatura, os quais abordam o tema, em sua maioria, em adultos e idosos^(^ ^19^ ^-^ ^22^ ^)^. Contudo, a busca na literatura pertinente mostrou escassez de estudos que analisem a modulação autonômica em crianças portadoras de DM1 utilizando a VFC como forma de análise. O DM1 leva a complicações irreversíveis ao organismo dessas crianças, prejudicando o crescimento em esfera comportamental, somática e social, além de diminuir a sensibilidade dos reflexos autonômicos, sendo em parte responsável pela redução da expectativa de vida em seus portadores^(^ ^23^ ^)^.

Dessa forma, este estudo teve como objetivo reunir informações atuais a respeito dos efeitos do DM1 sobre o comportamento autonômico cardíaco de crianças.

## Método

A busca pelos artigos utilizados neste estudo ocorreu de março a abril de 2013 por meio de pesquisas nas bases de dados *Medical Literature Analysis and Retrieval System Online* (Medline/PubMed), *Physiotherapy Evidence Database* (PEDro), *Scientific Electronic Library Online* (SciELO), *Literatura Latino-Americana e do Caribe em Ciências da Saúde* (Lilacs), *The Cochrane Library* (Cochrane) e Índice Bibliográfico Espanhol em Ciências da Saúde (Ibecs), considerando-se os artigos publicados de janeiro de 2003 a fevereiro de 2013.

Para a busca, utilizaram-se as seguintes palavras-chave: "sistema nervoso autônomo", "diabetes mellitus", "crianças", "diabetes mellitus tipo 1", "sistema nervoso simpático" e "sistema nervoso parassimpático". Os termos em língua portuguesa foram definidos com base nos Descritores em Ciências da Saúde (DeCS) e seus correspondentes em língua inglesa, no *Medical Subject Headings* (MeSH). Utilizou-se também nas buscas não o descritor, mas a palavra-chave deste estudo, "variabilidade da frequência cardíaca", e o seu correspondente em língua inglesa.

Com tais palavras, realizaram-se os seguintes cruzamentos: sistema nervoso autônomo *and* diabetes mellitus *and* crianças; diabetes mellitus *and* sistema nervoso autônomo; diabetes mellitus tipo 1 *and* sistema nervoso autônomo; diabetes mellitus *and* variabilidade da frequência cardíaca; diabetes mellitus *and* sistema nervoso simpático e diabetes mellitus *and* sistema nervoso parassimpático.

Como critérios de inclusão, consideraram-se os artigos publicados de janeiro de 2003 a fevereiro de 2013 em português, inglês e espanhol, abrangendo todos os tipos de desenho de estudo que utilizaram crianças com idade de nove a 12 anos com DM1 e que abordaram a influência do DM sobre o sistema nervoso autônomo, avaliada por meio da VFC. Excluíram-se da busca os trabalhos encontrados na forma de dissertações, teses e editoriais.

Para a seleção, os estudos passaram inicialmente por uma triagem dos títulos, realizada por um único pesquisador. Selecionaram-se os títulos que abordaram como ideia principal os seguintes aspectos: a influência do DM sobre o sistema nervoso autônomo, a VFC em crianças portadoras de DM, as consequências do diabetes na saúde dessas crianças e títulos que traziam evidências sobre o tema referido e as alterações que o DM promovia nos componentes simpáticos e parassimpáticos do sistema nervoso autônomo de crianças. Após essa seleção inicial, realizou-se uma filtragem na qual se excluíram os títulos repetidos, já que os artigos foram selecionados em diferentes bases de dados.

Em seguida, todos os títulos escolhidos tiveram seus resumos detalhadamente lidos e estudados a fim de selecionar os artigos que abordassem, em crianças, a influência do DM sobre o sistema nervoso autônomo, avaliada por meio da VFC. Após a exclusão dos resumos inadequados ao tema, avaliaram-se os textos completos e analisaram-se os que preencheram os critérios de inclusão. Além disso, todos os resumos selecionados tiveram suas referências verificadas para identificar os estudos que não foram encontrados na primeira busca eletrônica. Todas as etapas foram acompanhadas por um revisor sênior, que realizou o julgamento final de inclusão dos artigos.

Os dados foram descritos de forma qualitativa e tabulados conforme as seguintes características: autores e ano de publicação, objetivos, índices da VFC analisados e conclusões obtidas em cada artigo.

## Resultados

Na estratégia de busca e seleção eletrônica, localizaram-se 1.687 títulos. Dentre os artigos selecionados, eliminaram-se 1.495 referências que não abordavam o tema. Os 192 artigos restantes tiveram seus resumos lidos e, desse total, eliminaram-se mais 154 títulos por serem repetidos. Ao final, selecionaram-se 38 textos para análise integral, que levou à exclusão de outras 34 referências por não apresentarem a idade em questão, totalizando quatro artigos finais.

O Quadro 2 mostra os quatro artigos finais resultantes da busca eletrônica. Nenhum dos estudos encontrados foi randomizado ou semirrandomizado. Um dos estudos é observacional, descritivo e transversal^(^ ^24^ ^)^, dois são do tipo caso-controle, observacionais e transversais^(^ ^16^ ^,^ ^25^ ^)^ e outro é caso-controle, observacional e longitudinal^(^ ^26^ ^)^.

**Quadro 2:** Descrição dos estudos sobre a influên *Diferença estatística entre o Grupo Diabético e o Grupo Controle; **diferença estatística entre os grupos diabéticos VFC: variabilidade da frequência cardíaca; DM1: diabetes melito tipo 1; SDNN: desvio padrão de todos os intervalos R-R normais, expresso em milissegundos; SDANN: desvio padrão das médias dos intervalos R-R normais, a cada cinco minutos, em um intervalo de tempo, expresso em milissegundos; RMSSD: raiz quadrada da média do quadrado das diferenças entre intervalos R-R normais adjacentes, expressos em milissegundos; pNN50: porcentagem dos intervalos R-R adjacentes com diferença de duração maior que 50 milissegundos; ULF: componente de ultra baixa frequência; VLF: componente de muito baixa frequência; LF: componente de baixa frequência; nu: unidades normalizadas; HF: componente de alta frequência; LF/ HF: razão entre os componentes de baixa e alta frequência; TP: potência total em milissegundos ao quadrado (ms²); Hb1Ac: hemoglobina glicada

### Estudo observacional, descritivo e transversal

Chen *et al**^(^* ^24^ ^)^, utilizando os índices componente de baixa frequência - LF (em milissegundos ao quadrado - ms²), componente de alta frequência - HF (ms²) e potência total - TP (ms²), investigaram a influência do controle glicêmico (Hb1Ac), da duração do DM1 e do exercício físico sobre a modulação autonômica em crianças com DM1. As crianças analisadas foram divididas em quatro grupos: Grupo 1 (n=16; Hb1Ac ≤8%; idade: 9,6±1,0 anos; duração do DM1: 2,1±1,2 anos), Grupo 2 (n=15; Hb1Ac ≤8%; idade: 9,7±1,5 anos; duração do DM1: 6,5±1,4 anos), Grupo 3 (n=21; Hb1Ac >8%; idade: 10,1±1,2 anos; duração do DM1: 2,4±1,2 anos) e Grupo 4 (n=27; Hb1Ac >8%; idade: 10,6±1,4 anos; duração do DM1: 6,8±1,8 anos). Para participarem do estudo, as crianças não poderiam apresentar *Acantose Nigricans*, doenças hematológicas, síndrome de Turner e de Prader-Willi e também não poderiam tomar esteroides ou hormônios de crescimento.

Os autores observaram que, no repouso, a Hb1Ac e a duração da doença correlacionaram-se negativamente com todos os índices da VFC. Tanto a Hb1Ac quanto a duração da doença foram preditores significativos para o índice TP (ms²); entretanto, apenas a Hb1Ac foi um preditor significativo para os índices de LF (ms²) e HF (ms²). As crianças com DM1 que apresentaram Hb1Ac superior a 8% e duração da doença acima 4,5 anos tiveram uma menor VFC. Para o protocolo de exercício, as crianças ficaram dez minutos em estado de repouso e, em seguida, realizaram o exercício por dez minutos com um ritmo fixo em cima do *stepper* (JK-355c; JKexer, Taipei, Taiwan). O eletrocardiograma (ECG) foi monitorado continuamente durante todo o teste para se obterem os índices de VFC. Menores valores para todos os índices estudados foram observados durante o exercício em todos os grupos, indicando a não influência do controle glicêmico e da duração da doença sobre a VFC durante o exercício. 

### Estudos caso-controle, observacionais e transversais

Encontraram-se dois estudos desse tipo. Kardelen *et al**^(16) ^*investigaram, em crianças com DM1, a modulação autonômica por meio dos índices de VFC analisados nos domínios do tempo (SDNN, SDANN, RMSSD e pNN50) e da frequência (ULF, VLF, LF, HF e relação LF/HF). Analisaram-se os dados de 93 crianças distribuídas nos grupos Diabético (n=47; idade: 12,0±4,0 anos; duração do DM1: 4,2±3,2 anos) e Controle (n=46; idade: 10,8±3,1 anos). Para participar do estudo, as crianças não poderiam ter histórico de doença cardíaca e/ou respiratória, necessitar de medicação e ter doença ou consumo de medicamentos conhecidos por afetar a frequência cardíaca. 

Os autores observaram redução dos índices de VFC no domínio tanto do tempo, exceto para o SDANN, quanto da frequência, com redução acentuada nos valores de VLF, LF e HF. Os autores avaliaram também o ritmo circadiano da VFC e observaram diferenças entre os grupos. Todos os índices do tônus parassimpático e simpático avaliados durante um período de 24 horas aumentaram significativamente durante a noite. Nas crianças diabéticas com neuropatia autonômica, a demonstração da redução do aumento protetor do tônus parassimpático durante a noite levou à hipótese de que a predominância noturna da atividade simpática predispõe pacientes diabéticos a eventos cardiovasculares em qualquer momento. Essas alterações nos índices de VFC refletem uma significante redução da atividade parassimpática e, possivelmente, aumento da atividade simpática. Além disso, os autores observaram que a magnitude da influência do ritmo circadiano é menor nas crianças diabéticas em comparação às crianças normais.

Um artigo publicado por Chen *et al**^(^* ^25^ ^)^ explorou a influência do nível de atividade física, por meio de um questionário específico, sobre a função do sistema nervoso autônomo de crianças com DM1. As crianças analisadas foram divididas em: Grupo Diabético (n=93; idade: 10,3±1,6 anos; duração do DM1: 4,1±2,1 anos) e Grupo Controle (n=107; idade: 10,4±1,6 anos). Para participar do estudo, as crianças não poderiam ter evidências clínicas atuais ou pregressas de doença cardiovascular ou neurológica nem fazer uso de medicamentos que alterassem a função cardiovascular ou a atividade do sistema nervoso autônomo. Os resultados mostraram que, em repouso, os índices LH (ms²), HF (ms²) e TP (ms²) foram menores nos diabéticos que apresentaram baixa atividade física. As crianças com DM1 com atividade física de moderada a intensa não diferiram do grupo saudável. Os autores concluíram que crianças com DM1 devem ser estimuladas a praticarem atividades físicas, o que pode beneficiar a função do sistema nervoso autônomo.

### Estudo caso-controle, observacional e longitudinal

Lucini *et al**^(26) ^*analisaram a modulação autonômica de crianças e adolescentes com DM1 para avaliar as mudanças iniciais na regulação autonômica cardíaca e vascular e verificar a sua progressão ao longo do tempo. As crianças e os adolescentes foram divididos em quatro grupos: Grupo de Crianças Saudáveis (n=32; idade: 11,2±0,5 anos), Grupo de Crianças Diabéticas (n=46; idade: 11,5±0,4 anos; duração do DM1: 4,8±0,2 anos), Grupo de Adolescentes Saudáveis (n=36; idade: 20,2±0,3 anos) e Grupo de Adolescentes Diabéticos (n=47; idade: 19,3±0,2 anos; duração do DM1: 10,3±0,7 anos). Como critérios de inclusão, as crianças e adolescentes não poderiam: apresentar nenhuma doença concomitante (exceto o diabetes), usar medicação (exceto a insulina), fumar, beber ou ingerir quantidade abusiva de comida. Os autores mostraram que crianças e adolescentes com DM1 apresentam alterações autonômicas caracterizadas por diminuição barorreflexa e aumento de LF da pressão arterial sistólica, indicando uma menor VFC. Os autores também avaliaram essa população durante um ano e observaram progressão negativa nas alterações autonômicas, com redução barorreflexa e nos valores dos índices LF (em unidades normalizadas - nu) e HF (ms²).

## Discussão

Em geral, as análises dos textos selecionados demonstraram que: 1) crianças com DM1 apresentam redução da VFC global e da atividade vagal em comparação às crianças controle; 2) a prática de atividade física promove benefícios na modulação autonômica de crianças com DM1.

Corroborando os achados de Kardelen *et al**^(^* ^16^ ^)^, Jaiswal *et al*, em um estudo publicado em 2013, também observaram redução da VFC global em jovens com DM1 e média de idade de 18±8,0 anos^(^ ^27^ ^)^. Os autores sugerem que essa redução pode ser causada pela neuropatia autonômica diabética, a qual promove alterações dos processos fisiológicos do organismo, principalmente no sistema nervoso autônomo.

Maior VFC global relaciona-se a uma melhor condição do sistema nervoso autônomo e a maior eficiência das respostas aos estímulos tanto internos quanto externos ao organismo. Em contrapartida, a redução da VFC global, como a que ocorre em crianças com DM1, é um indicativo de adaptação anormal e insuficiente do sistema nervoso autônomo e de mau funcionamento fisiológico^(^ ^27^ ^,^ ^28^ ^)^. Verificou-se que a VFC é um método eficaz para detectar alterações autonômicas em crianças com DM1, permitindo melhor discriminação entre a fisiologia normal e anormal dessas crianças. A presença do DM1 pode levar à perda da qualidade de vida, ao aumento do risco de morbidade e mortalidade em decorrência de alterações relevantes do sistema nervoso autônomo e à exposição prolongada a elevados níveis glicêmicos^(^ ^29^ ^)^. 

Considerando as diversas alterações promovidas pelo DM1 em crianças, destaca-se a importância do tratamento adequado na busca de condições saudáveis de desenvolvimento, crescimento e prevenção de comorbidades. Nesse contexto, a prática de atividade física - que proporciona melhora na captação de glicose pelos tecidos independentemente da insulina, aumento da permeabilidade da membrana citoplasmática e potencialização da ação do hormônio, com redução da quantidade de medicação e das complicações ocasionadas pelo DM^(^ ^30^ ^)^ - mereceu destaque na literatura. O artigo publicado por Chen *et al**^(^* ^25^ ^)^ explorou a influência do nível de atividade física sobre a função do sistema nervoso autônomo de crianças com DM1. Para avaliar o nível de atividade física, os autores utilizaram um questionário específico; contudo, estudos que avaliem o efeito da atividade física sobre o condicionamento cardiovascular de forma direta e objetiva são necessários para confirmar a importância da prática no controle autonômico nessa população. 

Outros resultados, também publicados por Chen *et al**^(^* ^24^ ^)^ envolvendo o exercício físico, mostraram a não influência do controle glicêmico e da duração da doença sobre a VFC durante o exercício. Entretanto, os indivíduos com DM1 que apresentaram Hb1Ac superior a 8% e duração da doença acima 4,5 anos tiveram uma menor VFC. A utilização de apenas um teste de Hb1Ac como indicador de controle glicêmico pode ser considerada uma limitação do estudo.

Para finalizar, algumas considerações merecem destaque. Nenhum dos estudos encontrados foram ensaios clínicos randomizados, bem como não foram combinados na forma de metanálise, o que demonstra a baixa evidência científica da temática e abre perspectivas para estudos na área. Quanto aos aspectos metodológicos dos estudos analisados, não se descreveram as condições ambientais (como temperatura e umidade) em que foram realizadas as avaliações autonômicas e não há menção se as crianças analisadas realizavam algum tipo de exercício físico com regularidade. 

Em resumo, os trabalhos demonstraram que crianças com DM1 apresentam modificações na modulação autonômica caracterizadas pela redução global da VFC e da atividade vagal. Além disso, deve-se ressaltar que a prática de qualquer atividade física foi benéfica ao organismo dessas crianças, pois promoveu aumento da atividade vagal.
